# Supplementary material for: Saccharomyces boulardii (CNCM I-745) improves intestinal damage in sepsis by remodeling intestinal flora structure
Source: Front Cell Infect Microbiol. 2025 Jul 31;15:1602792. doi: 10.3389/fcimb.2025.1602792 (PMC12350329; doi:10.3389/fcimb.2025.1602792)
Supplement: Supplementary file 1 [file DataSheet1.zip › summary/summary/8_Advanced_analyse/2_PICRUSt2_result/KEGG/Group.BW0_vs_AW0/PICRUSt2_KO_Group.BW0_vs_AW0_diff.pdf]

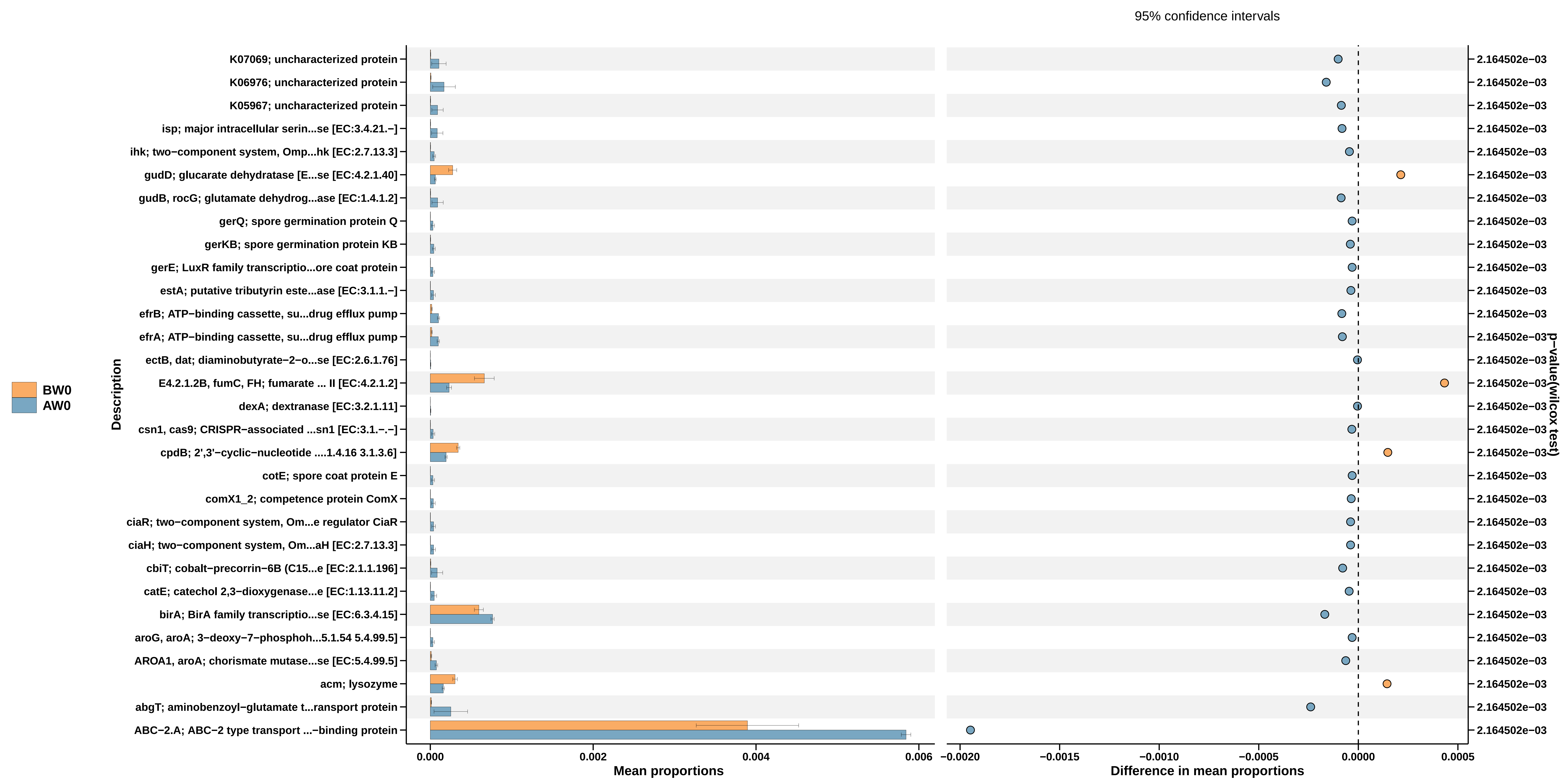
95% confidence intervals
K07069; uncharacterized protein
K06976; uncharacterized protein
K05967; uncharacterized protein
isp; major intracellular serin...se [EC:3.4.21.−]
ihk; two−component system, Omp...hk [EC:2.7.13.3]
gudD; glucarate dehydratase [E...se [EC:4.2.1.40]
gudB, rocG; glutamate dehydrog...ase [EC:1.4.1.2]
gerQ; spore germination protein Q
gerKB; spore germination protein KB
gerE; LuxR family transcriptio...ore coat protein
estA; putative tributyrin este...ase [EC:3.1.1.−]
efrB; ATP−binding cassette, su...drug efflux pump
efrA; ATP−binding cassette, su...drug efflux pump
ectB, dat; diaminobutyrate−2−o...se [EC:2.6.1.76]
E4.2.1.2B, fumC, FH; fumarate ... II [EC:4.2.1.2]
dexA; dextranase [EC:3.2.1.11]
csn1, cas9; CRISPR−associated ...sn1 [EC:3.1.−.−]
cpdB; 2',3'−cyclic−nucleotide ....1.4.16 3.1.3.6]
cotE; spore coat protein E
comX1_2; competence protein ComX
ciaR; two−component system, Om...e regulator CiaR
ciaH; two−component system, Om...aH [EC:2.7.13.3]
cbiT; cobalt−precorrin−6B (C15...e [EC:2.1.1.196]
catE; catechol 2,3−dioxygenase...e [EC:1.13.11.2]
birA; BirA family transcriptio...se [EC:6.3.4.15]
aroG, aroA; 3−deoxy−7−phosphoh...5.1.54 5.4.99.5]
AROA1, aroA; chorismate mutase...se [EC:5.4.99.5]
acm; lysozyme
abgT; aminobenzoyl−glutamate t...ransport protein
ABC−2.A; ABC−2 type transport ...−binding protein
Description
BW0
AW0
0.000
0.002
0.004
0.006
Mean proportions
−0.0020
−0.0015
−0.0010
−0.0005
0.0000
0.0005
Difference in mean proportions
p−value(wilcox test)
2.164502e−03
